# TRAUMA OBSCURING LEUKEMIA - A UNIQUE CASE REPORT

**DOI:** 10.51894/001c.123025

**Published:** 2024-08-30

**Authors:** Celine Adriano

**Affiliations:** 1 College of Osteopathic Medicine Michigan State University

## 56

### INTRODUCTION

Acute myeloid leukemia is a malignancy of the myeloid cell line commonly affecting adults. Risk factors include exposure to benzene, chemotherapeutics, and Down syndrome. We report a case of a 68-year-old patient with persistent symptoms of dyspnea, fatigue, and bruising. Although the patient had a prior workplace injury potentially exacerbating these symptoms, diagnostic workup revealed an underlying myelodysplastic syndrome.

### CASE DESCRIPTION

A 68-year-old patient with a past medical history of hypertension, seizure disorder, and dementia presented to the emergency department with a chief complaint of generalized fatigue. He worked as a mechanic and suffered a traumatic injury three months prior. On physical examination, the patient had left-sided contusions and diffuse ecchymoses. The patient visited several urgent care facilities due to these symptoms, which were attributed to the prior injury. Diagnostic workup included a complete blood count, a basic metabolic panel, an electrocardiogram, a CT angiogram of the chest, abdomen, and pelvis, and a chest X-ray. EKG and imaging studies demonstrated no acute process. CBC was significant for a hemoglobin of 7.0, a white blood cell count of 25.73, and a platelet count of 22,000. These findings prompted hospital admission to perform a bone marrow biopsy. Aspirate showed bone marrow consistent with high-grade myelodysplastic syndrome evolving into acute myeloid leukemia.

### DISCUSSION

Acute myeloid leukemia is a cancer of hematopoietic stem cells. The pathophysiology is related to myeloblast cells proliferating to the point where the bone marrow cannot function, leading to symptoms of pancytopenia. Considering the ambiguity of its presentation, AML is often first detected on routine blood work. A confirmatory diagnosis is made with a bone marrow biopsy. Although treatments will vary based on the subtype of AML, systemic chemotherapy remains the mainstay of treatment. Patients diagnosed have a poor prognosis, with a five-year survival rate of 28.3%.

### CONCLUSION

At its core, this is a case of missed diagnosis and the need to emphasize holistic workup. Although this patient experienced a traumatic event that could have accounted for his symptoms, he was seen by several facilities without thorough care, causing delay in the diagnosis and treatment.

